# Comparison of Short vs Long Anti-rotation in Treating Trochanteric Fractures

**DOI:** 10.5704/MOJ.1603.005

**Published:** 2016-03

**Authors:** P Raval, A Ramasamy, H Raza, K Khan, N Awan

**Affiliations:** Department of Orthopaedics, Wishaw General Hospital, Scotland, United Kingdom

**Keywords:** Pertrochanteric fractures, Proximal Femoral Nail Antirotation, Short, Long, Intramedullary fixation

## Abstract

**Introduction**: A comparative evaluation of the surgical treatment and outcome of patients with pertrochanteric fractures treated with short versus long proximal femoral nail antirotation.

**Materials and methods**: A retrospective review was conducted of patients with pertrochanteric fractures treated between January 2011 and June 2012. In all 80 patients were enrolled in the study, of which 40 were treated with short PFNA and the remaining with long PFNA. Comparative analyses of demographic data, peri-operative outcome and complications were carried out.

**Results**: There was no significant difference noted in the two groups with regards to Arbeitsgemeinschaft fur Osteosynthesefragen (AO) fracture classification, time from injury to surgery, blood transfusion post surgery and hospital stay. The surgical duration for a short PFNA procedure was significantly less (58 minutes) when compared to that of a long PFNA (87 minutes). Similarly intra-operative blood loss was significantly higher in the long PFNA group as compared to the short PFNA.

**Conclusions**: A relatively quicker surgical time of just under an hour , lesser blood loss and better learning curve with trainee surgeons make short PFNA a better implant choice in the treatment of pertrochanteric fractures.

## Introduction

The advancement in modern medicine has enabled many to live long. However, as the age advances so do certain comorbidities. Osteoporosis is one such significant comorbidity. The increased prevalence of hip fractures in osteoporotic individuals is well known^1,2^. Trivial trauma such as a fall from a standing height is the most common cause for sustaining a fragility fracture^3^. The number of hip fractures has been estimated to rise over the next few years, leading to increasing costs and subsequent rising financial burden^4^.

Pertrochanteric fractures are those which are around the trochanteric region of femur. They are classified as 31-A1 pertrochanteric simple, pertrochanteric multifragmentary and 31-A3 intertrochanteric as per the Arbeitsgemeinschaft fur Osteosynthesefragen (AO) classification^5^.

There has been considerable debate over the method of treating pertrochanteric fractures. Meta analytical studies in literature have not been able to come to a consensus whether an extramedullary or an intramedullary implant is better in this regard^6-8^.

In our institution pertrochanteric fractures are treated by using intramedullary devices, such as the AO-Proximal Femoral Nail Antirotation (AO PFN-A). The PFN-A is available in various sizes. The short version has 170mm, 200mm, 240mm as length and the long version if from 300-420mm with 20mm increments and a bending radius of 1500mm^9^. Both the short and the long PFN-A implants are routinely employed in the surgical treatment of pertrochanteric fractures at our hospital.

The purpose of this retrospective study is to do a comparative evaluation of the surgical outcome of patients with pertrochanteric fractures treated using either a short or a long PFN-A.

## Materials and Methods

Retrospective data was collected from the operation theatre implant book regarding the type of PFN-A used. Using that data the relevant patient charts were retrieved from the medical records. A total of 117 patients underwent surgery for pertrochanteric fracture using PFN-A, between January 2011 and June 2012. However only 80 patients met the inclusion criteria for the study (Table I). Patients with multiple injuries and pathological fractures were excluded from the study.

**Table I:** Patient selection criteria

Inclusion Criteria	Exclusion Criteria
• Patients with age > 60 years	• Polytrauma patients
• Pertrochanteric fracture sustained after trivial trauma	• Patients with pathological fractures
	• Patients with concomitant sub-trochanteric or shaft femur fractures

The surgery was performed as soon as the clinical condition of the patient permitted (Table II). All patients were started on mechanical and chemical methods of deep venous thrombosis (DVT) prophylaxis on admission, as per the hospital guidelines. Antibiotics belonging to the beta-lactam group were administered 30 minutes prior to incision and two doses were repeated post surgery. In cases of penicillin allergy, macrolide or quinolone group of antibiotics were used. Spinal anaesthesia was administered in majority of the cases (Table III).

**Table II:** Pre-operative Data (Demographics, Fracture type, Time to surgery)

Variable	Total	Short PFN-A	Long PFN-A	*‘p’* value
Number	80	40	40	–
Gender				
Male/Female	23/57	11/29	13/27	0.625^$^
Age(years)				
Mean +/− SD	75.9 +/− 9.1	77.1 +/− 9.2	76.1 +/− 8.7	0.806^£^
AO fracture type				
31-A1/A2/A3	21 / 48 / 11	12 / 24 / 4	9 / 24 / 7	0.536^$^
Time between injury and surgery (days)				
Mean +/− SD	2.3 +/− 1.9	2.2 +/− 2.2	2.5 +/− 1.7	0.744^£^

^$^ Chi-square test

^£^ Two sample t-test

**Table III:** Intra-operative Data (Anaesthesia, Duration, Blood loss, Operating surgeon)

Variable	Total	Short PFN-A	Long PFN-A	‘p’ value
Number	80	40	40	--
Anaesthesia				
(General/Spinal)	21 / 59	12 / 28	9 / 31	0.445^$^
Duration of surgery (minutes)				
Mean +/− SD	72.3 +/− 28.0	58.6 +/− 12.6	87.7 +/− 32.6	0.016^£^
Open reduction of fracture (n)	----	1	4	----
Intra-operative blood loss (ml)				
Mean +/− SD	253.1 +/− 190.6	172.7 +/− 156.9	341.7 +/− 191.8	0.042^£^
Operating Surgeon				
Consultant / Registrar under supervision	64 / 16	28 / 12	36 / 4	0.025^$^

^$^Chi-square test

^£^Two sample t-test

Our institution is a major teaching hospital and most surgeries in this particular study were performed by the Consultant Orthopaedic surgeon, while some were performed by senior trainee residents under direct supervision of the consultant. Surgeries were performed on a traction table under the guidance of an image intensifier. Longitudinal traction was applied to achieve reduction and realignment of the fracture fragments. Open reduction was resorted to when the closed manoeuvre failed. Distal locking was performed using the aiming device for the short PFN-A, whereas a free hand technique was employed for the long PFN-A. Reaming was performed in all cases. The standard operating manual as supplied by the manufacturer was followed during the procedure^9^.

Intraoperatively, the duration of surgery, method of fracture reduction (closed versus open) and blood loss were recorded (Table III). Postoperative radiographs were taken to ascertain the fracture reduction and position of the implant. Patients were started on active and passive movements immediately and partial or full weight bearing was allowed as soon as the patient’s general condition and any pre-existing comorbidity allowed. Subcutaneous DVT prophylaxis was continued till the patient was satisfactorily mobile. Any blood transfusions and total days of hospitalisation were noted (Table IV). Patients were discharged on oral anticoagulants.

**Table IV:** Post-operative Data (Transfusion, Hospital stay, Reoperations, Mortality)

Variable	Total	Short PFN-A	Long PFN-A	‘p’ value
Number	80	40	40	--
Blood transfusion (n)	12 / 80	4 / 40	8 / 40	0.210^$^
Hospital stay (days)	11 +/− 5.4	11.1 +/− 6.2	10.9 +/− 4.8	0.937^£^
Reoperations(n)	3 / 80	1 / 40	2 / 40	0.556^$^
Mortality (n)	8 / 80	3 / 40	5 / 40	0.456^$^

^$^ Chi-square test

^£^ Two sample t-test

All patients were monitored at 1, 3, 6 and 12 months after the surgery. Radiographs were taken at each follow up and compared against the previous images. At each visit, status of fracture union was noted. All patients had radiological union evident by the 12th month of follow up. Improvement in mobility of patients was noted during each visit.

**Fig. 1:**
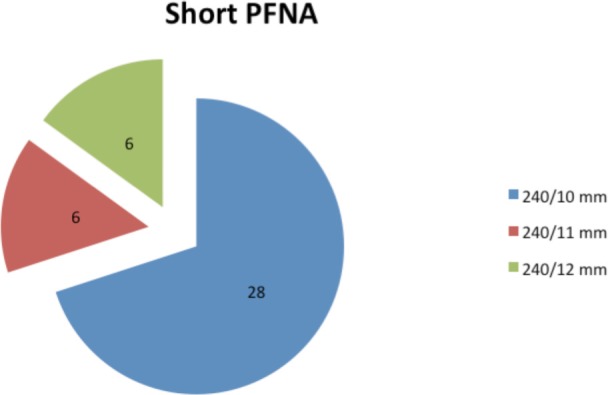
Dimensions of Short PFN-A.

**Fig. 2:**
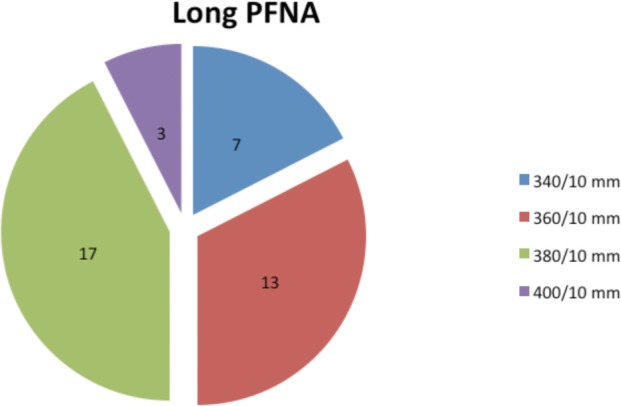
Dimensions of Long PFN-A.

All implants used in the short PFN-A group had a length of 240mm but different diameters. (Figure 1). However implants of varying lengths were used in the long PFNA group (Figure 2).

Statistical analyses were conducted using Analysis ToolPak on Microsoft Excel 2010 (v14.0). Continuous normally distributed data were analysed using a two sample Student’s t test and Pearson’s chi-square test was used to compare groups with categorical variables. A probability (p value) of < 0.05 was considered to be statistically significant.

## Results

80 patients underwent surgery for pertrochanteric fractures in the period from January 2011 and June 2012. There were 40 patients each in the short and long PFNA groups. The patient characteristics of both groups was not significantly different (Table II)

Both groups had a higher number of female patients with an average of around 70 %. The mean age for the short PFNA group was 77 years (range: 68-86 years) and 76 years (range: 68-84 years) for the long PFNA group. There were identical numbers of 31-A1 fractures in both groups and this was also the most common fracture type in either. Most patients in both groups were operated upon within three days of sustaining the injury.

There was no significant difference in the type of anaesthesia administered to the patients in either groups (p = 0.445). Spinal anaesthesia however, was most commonly administered. 70 % of the short PFNA patients and 77 % of the long group got spinal anaesthesia.

30 % of the short PFNA surgeries were performed by trainee residents as compared to only 10 % of the long PFN-A ones (p = 0.025). These were performed under direct supervision of the Consultant Orthopaedic surgeon. Most procedures were performed closed, however one open reduction was performed in the short PFN-A group and four were performed in the other group.

The amount of blood loss was greater in the long PFN-A group (341ml vs. 172ml, p = 0.042). The operative procedure lasted for 58 minutes (range: 46-70 minutes) in the short PFN-A group as compared to 87 minutes (range: 55-119 minutes) in the long PFN-A group (p = 0.016) (Table III).

There was no significant difference in the postoperative outcome of both the groups and the data was comparable (Table IV). Twice the number of patients (eight vs four) got blood transfusions in the long PFN-A group as compared to the other. The mean hospital stay for both groups was similar at approximately 11 days. None of the patients of either group had any infection while they were in the hospital. All patients were discharged once they had independent mobility and their assigned physical therapists were satisfied with their progress.

Three patients had reoperations. Two from the long PFN-A group and one from the short. There was a screw cut-out in the short PFN-A patient, which was subsequently revised to a hemiarthroplasty. Of the other two patients in the long PFN-A group, one sustained a fracture at the distal end of the nail. This was treated by using a locking plate. The remaining patient had a perforation of the anterior femoral cortex. In retrospect this failure was attributed to multiple failed attempts at distal locking, which however created a stress riser at that point. A retrograde nailing was performed for this patient.

All patients were followed up for one year. Five patients (12.5 %) in the long PFN-A group and three patients (7.5 %) in the short PFN-A group had died within one year of undergoing the surgery (p = 0.456).

## Discussion

Hip fractures are a serious cause of concern in the osteoporotic elderly population. The associated mortality and morbidity with hip fractures is significant^2^. An everincreasing aged population only compounds this problem. The number of fragility hip fractures is expected to rise exponentially with time and so are the corresponding costs^4^. Almost 90% of hip fractures are sustained after having a fall^10^. It would be wonderful if one could identify a particular group of elderly patients who were vulnerable to sustain fragility fractures around the hip. It has been shown that bone mineral densitometry (BMD) of the trochanteric region of femur, obtained using a dual energy X-ray absorptiometry (DXA), is the best indicator for predicting pertrochanteric fractures^11^. The mean force required to cause a pertrochanteric fracture is 3107+/−1066 N, however this value was arrived at, without considering other factors such as fall biomechanics and concomitantly acting muscular forces around the hip joint^11^.

A finite element (FE) model study of femur has shown pertrochanteric fractures to generate a stress of 621MPa. The largest stress concentration is at the lag screw hole of the intramedullary implant. Similarly interfragmentary (IFM) movements between the proximal and distal fracture fragments in both axial and transverse direction were highest in the FE model with pertrochanteric fracture. Such large forces are responsible for sliding of the fracture fragments on one another, subsequently leading to opening up of the fracture. The angle of insertion of a nail during surgery also is an important factor, since the pre-stress of the nail depends on the angle of insertion^12^.

Lag screw cut out in the treatment of pertrochanteric fractures is well documented^13-15^. Ideal lag screw placement should have a tip-apex distance of less than 25mm to avoid a screw cut out^16,17^. In a cadaveric study it has been demonstrated that multiplanar cyclic loading caused the femoral head to rotate. Eccentrically placed lag screws cause both rotational cut out and varus collapse. Around 12 % of pertrochanteric fractures undergo progressive rotation as they collapse and rotation has been shown to more common in cases with lag screw out^18,19^. The Proximal Femoral Nail Antirotation (PFN-A) , as the name implies, has an inbuilt mechanism which negates the aforementioned rotational effect. In our study only one patient had a screw cut and who subsequently underwent a hemiarthroplasty.

Patients who underwent short PFN-A procedures in the current study had lesser bleeding as compared to the long PFN-A group. This was a significant finding (p=0.042). It has been shown that intramedullary fixation procedures lead to a larger blood loss as compared to extramedullary fixation. Proximal reaming and insertion of a longer nail leading to opening of the medullary canal leads to increased blood loss. Most of the time, such a blood loss is concealed^20^.

The correct technique of PFN-A insertion can avoid the operating surgeon a lot of grief. Practical suggestions given by Hwang *et al.*, are noteworthy^21^. The ethnic background of the patient should be borne in mind while operating, especially the Asian population. An excessive anterior bow in a relatively shorter femur should be paid special attention^22^. The nail entry point has to be precise. Longer nails are recommended in elderly patients with significant osteoarthritis, because the entry point is more anatomically aligned as compared to the short nails. The operating surgeon is advised to refrain from hammering the nail in, however gentle the hammering process may be^21^.

One of the initial studies done in European clinics on the efficacy of PFN-A, found it to be an ideal implant in the treatment of unstable pertrochanteric fractures. This was especially in regard to the prevention of femoral head penetration by the screw. Ipsilateral femoral shaft fractures, due to missed attempts at distal locking have been reported in this study^23^. Multiple failed attempts at distal locking, especially when performed free hand not only increase the total operative time but also create stress risers in the femoral shaft, which can be sites for potential implant failures in the future. Authors of the current study had an identical experience. A retrograde nailing was done in that particular case. A subsequent study comparing the placement of a single fixation device in the femoral head in PFN-A did not confer any additional advantage to that of a PFN (Proximal Femoral Nail) in which an additional screw is placed proximally^24^.

In comparison between the efficacies of PFN-A with Dynamic Hip Screw (DHS), it has been convincingly reported that a PFN-A is a better implant. Not only is the PFN-A a biomechanically superior implant, but also the surgical time and fluoroscopy exposure is less, with fewer complications. Patients with PFN-A mobilised earlier than those who had a DHS^25,26^. As regards to mortality, a meta-analytical study showed no difference in prognosis in either groups^26^. A recent study comparing PFN-A with DCS has reported a similar outcome, in favour of the PFN-A^27^.

Aguado-Maestro*et al.* , in their recent study of 200 patients of pertrochanteric fractures treated with PFN-A, state that the helical blade system reduced the rate of cut through and cut out in pertrochanteric fractures and accurate placement of the helical blade was a key parameter to avoid mechanical failures^28^. A possible theoretical explanation of the helical blade failure has been attributed to torsional forces generated during ambulation. These forces cause a peri implant osteolysis, subsequently leading to implant failure. This phenomenon has been named as a “windshield effect”^29^.

Computer navigation has opened newer avenues in the field of Orthopaedics. In an experimental study of computer assisted planning and navigation, the operating surgeon is guided to stay inside a three dimensional ‘safe zone’ while inserting the helical screw. If inadvertently the surgeon leaves the safe zone, a warning message, ‘perforation’ is displayed by the software. Although the fluoroscopic images and drilling attempts were significantly reduced by using a computer guided system when compared to a minimally invasive procedure, there was no difference noted between a computer guided and an open procedure. Other significant issue noted was that of a longer surgical duration in a computer navigation assisted procedure.

One limitation of this study is that many different surgeons operated upon the patients of either group. This could be a confounding factor. However the authors of this study can state that all surgeons involved were very well versed with the operative technique of PFN-A. There was nobody in the group who was a novice. Overall the short PFN-A was favoured by the trainees because of a relatively easier operative technique. This was shown to be statistically significant (p = 0.025) (Table III).

Why certain surgeons preferred a long PFN-A for a pertrochanteric fracture when the standard recommendation was a short PFN-A, this was one question which had no satisfactory answers. Informal discussion with the operating surgeons who preferred a longer nail, revealed that an anticipation of a sub-trochanteric extension of a seemingly normal inter-trochanteric fracture forced them to be ‘safe than sorry’. Now whether additional investigations like a magnetic resonance imaging (MRI) or a (computed tomography) CT scan are warranted to see for such subtrochanteric extensions is an entirely new topic of discussion as per the authors of the current study.

## Conclusion

The treatment of Pertrochanteric fractures will continue to be a challenge to the treating surgeon. Current literature is in favour of using an intramedullary device like a PFN-A. The authors of this particular study would recommend using a short PFN-A, on account of lesser duration of surgery, hence lesser anaesthesia time in older patients and also lesser blood loss. Relatively shorter learning curve amongst trainee surgeons is another reason why a short PFN-A should be given a preference over a long PFN-A. However, past learning experiences will play a significant role in choosing the correct length of the implant.

## Conflict of Interest

The authors declare that they have no competing interests.
